# SIMILE enables alignment of tandem mass spectra with statistical significance

**DOI:** 10.1038/s41467-022-30118-9

**Published:** 2022-05-06

**Authors:** Daniel G. C. Treen, Mingxun Wang, Shipei Xing, Katherine B. Louie, Tao Huan, Pieter C. Dorrestein, Trent R. Northen, Benjamin P. Bowen

**Affiliations:** 1 Environmental Genomics and Systems Biology Division & The Joint Genome Institute Lawrence Berkeley National Laboratory, One Cyclotron Road, Berkeley, CA 94720 United States; 2 Collaborative Mass Spectrometry Innovation Center, Skagss school of Pharmacy and Pharmaceutical Sciences, Departments of Pharmacology and Pediatrics, University of California, San Diego, 9500 Gilman Drive, La Jolla, CA 92093 United States; 3 Department of Chemistry, Faculty of Science, University of British Columbia, Vancouver Campus, 2036 Main Mall, Vancouver, V6T 1Z1 BC Canada

**Keywords:** Metabolomics, Cheminformatics, Computational models

## Abstract

Interrelating small molecules according to their aligned fragmentation spectra is central to tandem mass spectrometry-based untargeted metabolomics. Current alignment algorithms do not provide statistical significance and compounds that have multiple delocalized structural differences and therefore often fail to have their fragment ions aligned. Here we align fragmentation spectra with both statistical significance and allowance for multiple chemical differences using Significant Interrelation of MS/MS Ions via Laplacian Embedding (SIMILE). SIMILE yields spectral alignment inferred structural connections in molecular networks that are not found with cosine-based scoring algorithms. In addition, it is now possible to rank spectral alignments based on p-values in the exploration of structural relationships between compounds and enhance the chemical connectivity that can be obtained with molecular networking.

## Abstract (web summary)

Interrelating metabolites by their fragmentation spectra is central to metabolomics. Here the authors align fragmentation spectra with both statistical significance and allowance for multiple chemical differences using Significant Interrelation of MS/MS Ions via Laplacian Embedding (SIMILE).

## Introduction

Tandem mass spectrometry is widely used in metabolomics experiments to hypothesize chemical structures. This is done by aligning fragment ions that share the same mass-to-charge ratio (*m/z*) and calculating the cosine similarity of their intensities^1^. Such compound identification often requires determining if an experimental fragmentation spectrum matches an authentic standard with the annotated data.

Recently, alignment approaches have been developed that aim to yield scores that are a proxy for compound similarity rather than identity. For instance, GNPS-based molecular networking and NIST Hybrid Search both implement an alignment approach that is sensitive to compounds that differ by a single/localized structural difference(s)^2–4^. The general logic for these two approaches is as follows: when a pair of related molecules are fragmented, their fragmentation data are likely similar. Under the assumption that the difference in masses stems from a single/localized structural difference and does not alter the fragmentation process of the molecule, the structural difference can either be attached to charged fragments or localized modifications that are reflected in neutral mass additions in the fragment ions (e.g., a lipid may have an additional mass of 14, 26, or 28 Da representing CH_2_, CH=CH, or CH_2_–CH_2_ additions). The charged fragments are directly observed as *m/z’*s in the fragmentation spectrum, while the neutral fragments can be indirectly observed as neutral losses by subtracting (or adding) the fragment *m/z’*s from their precursor *m/z*. Therefore, when the assumptions hold as is often the case, two fragments from different molecules can be aligned if they share the same *m/z* or the same *m/z* difference with respect to their precursor *m/z*. More recently, a concept of hypothetical neutral loss is proposed to further align neutral losses from pairs of fragment ions, showing significantly improved correlation between spectral and structural similarities^5^. Alignment approaches on fragmentation data have also proven useful for mass spectrometry-based proteomics by identifying pairs of peptides that differ by multiple modifications^6–8^.

Machine-learning approaches such as SIRIUS, CANOPUS, MS2LDA, and Spec2Vec also incorporate precursor ion neutral losses as a feature in their implementations^9–12^. Recent implementations combining machine learning with in silico structural database searching allow exploring high-confidence identifications to explore biochemistry outside of known chemical databases^13^. Other tools have enabled the false-discovery rates from tandem mass spectra database searches to separate correct from incorrect hits through false-discovery rate assignments (analogous to decoy database searching in proteomics). While there are methods for estimating statistical significance for compound identification, to our knowledge, no method for calculating the significance of fragmentation-spectra alignments from a pair of spectra has been described^14,15^.

Protein-sequence alignment algorithms like Needleman–Wunsch, Smith–Waterman, and BLAST yield alignments with statistical significance that are robust to multiple substitutions, insertions, and deletions^16,17^. These methods are fundamentally different from fragmentation-spectra-based cosine similarity in that they rely on substitution matrices describing the log odds of amino acids sharing common ancestry relative to random chance such as the PAM and BLOSUM matrices^18,19^. These approaches have not been widely applied to fragmentation data for two reasons: first, unlike protein- substitution matrices that are generally of size 20 by 20 (amino acids), a global substitution matrix for fragment ions would be infinite due to the infinite number of possible *m/z* values; and second, *m/z* values are only partially tied to chemical structure due to the one-to-many correspondence between *m/z* values and chemical structures. However, if restricted to a single pair of fragmentation spectra, a spectral graph-theoretic framework parameterized by their all-by-all *m/z* difference counts can generate finite, context sensitive, and mathematically consistent fragment ion similarity matrices based on average commute times^20^.

**Fig. 1:**
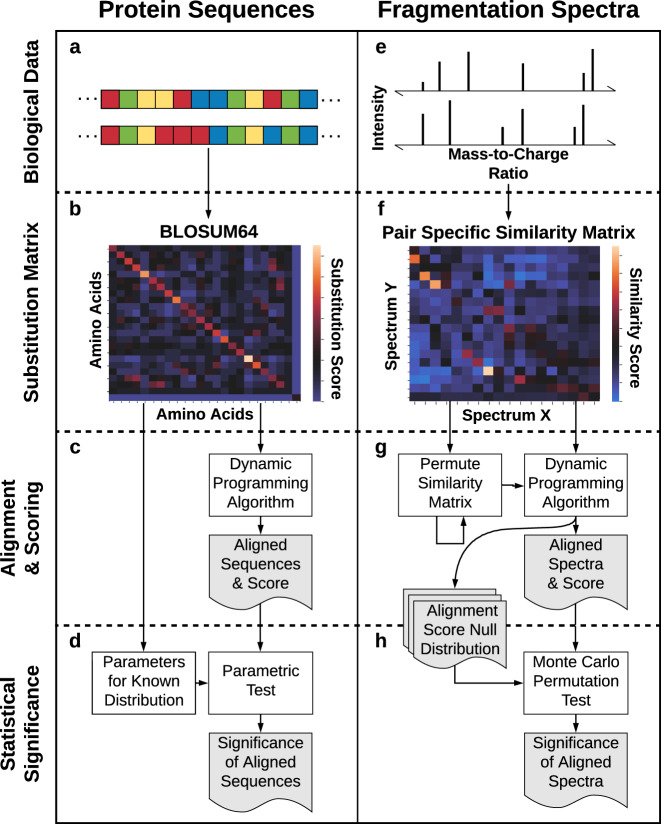
Analogous to how protein sequences undergo alignment, SIMILE aligns fragmentation spectra with allowances for substitutions and gaps. **a**–**c** Pairs of protein sequences are globally aligned by using a dynamic programming algorithm such as Needleman–Wunsch with an evolutionary model-based substitution matrix and gap penalty. **d** Significance of the alignment is calculated by assuming alignment scores that follow a distribution parameterized by choice of substitution matrix. **e**–**g** Likewise, pairs of fragmentation spectra are aligned by a dynamic programming algorithm with a pair-specific similarity matrix and a gap penalty of zero. **g**, **h** The alignment-score null distribution used to calculate the significance of observed alignments stems from permuting pair-specific similarity matrix interrelating fragmentation spectra X and Y randomly many times with restrictions according to the null hypothesis.

**Fig. 2:**
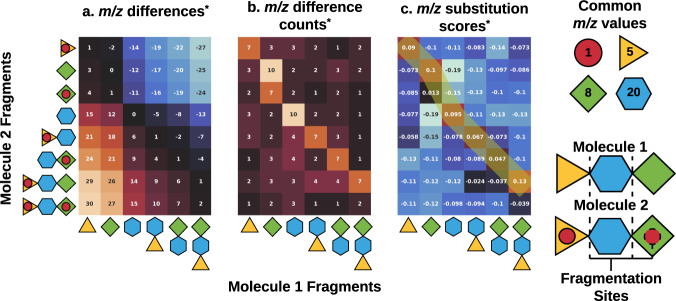
For two hypothetical molecules, this cartoon illustrates the underlying steps of the SIMILE algorithm in calculating pairwise *m/z* similarity matrices. For this example, two molecules “1” and “2” are shown. Each molecule is made from three blocks and there are two modifiers to molecule 2. **a** All pairwise *m/z* differences between fragments of molecule 1 and molecule 2 are stored in a matrix. **b** All entries with the same *m/z* difference in the top-two quadrants are replaced by the number of entries in which that *m/z* difference occurred. The same process is repeated for the bottom-two quadrants. **c** Calculating the pseudoinverse of the directed graph laplacian with this matrix yields similarity scores for each pair of *m/z* values. The quadrants interrelating molecules 1 and 2 can then be fed into a dynamic programming algorithm to yield aligned fragment ions between molecules 1 and 2. For illustrative purposes, only the quadrant corresponding to molecule 1 vs. molecule 2 of the full matrices is shown. The other quadrants corresponding to molecule 1 vs. molecule 1, molecule 2 vs. molecule 1, and molecule 2 vs. molecule 2 are still used for calculations.

Here, we introduce Significant Interrelation of MS/MS Ions via Laplacian Embedding (SIMILE), an approach that leverages methods used for protein-sequence alignment to enable robust pairwise alignment of fragmentation spectra with *p*-value estimation (Fig. 1). Rather than requiring identical *m/z* values or precursor ion neutral losses for alignment of fragmentation spectra, SIMILE uses all *m/z* differences among a pair of fragmentation spectra to generate a pair-specific fragment ion similarity matrix. This matrix is then used as the input to a dynamic programming alignment algorithm for alignment and scoring. The significance of an alignment is calculated via a Monte Carlo permutation test with alignment score as the test statistic under the null hypothesis that *m/z* values are exchangeable between *m/z-*ordered fragmentation spectra only if they yield hypothetical fragmentation spectra that are also *m/z* ordered. Figure 2 illustrates these aspects of the SIMILE algorithm with two hypothetical molecules.

## Results

### SIMILE benchmarking

We used tandem mass spectra from NIST20’s Small Molecule High Resolution Accurate Mass MS/MS Library to compare SIMILE to existing algorithms, determine strengths of SIMILE, and identify potential areas for improvement of SIMILE. The reference tandem mass spectra of compounds were filtered by unique InChIKey closest to 40-eV collision energy within ± 5 eV, resulting in a dataset comprising a single spectra for each of 24.5k molecules (7356 molecules as negative-mode [M–H]− adducts and 17,225 molecules as positive-mode [M + H] + adducts). These spectra were used in analysis without modification or further filtering.

We compared “pairs of spectra” from the spectral dataset, and evenly sampled 100,000 pairs from each Classyfire compound class, with the requirement that at least one compound in each pair was from a specific compound class. Compound classes with less than 100,000 pairs were eliminated, resulting in 63 unique compound classes for negative ionization mode and 145 unique compound classes for positive ionization mode having at least 100,000 pairs for comparison. Rather than focusing on performance across compound classes, this approach simply served to remove unexpected bias in the dataset where a small number of compound classes might be overrepresented. This gave a final total for negative ionization mode of 6,300,000 pairs and for positive ionization mode 14,500,000 pairs for analysis.

**Fig. 3:**
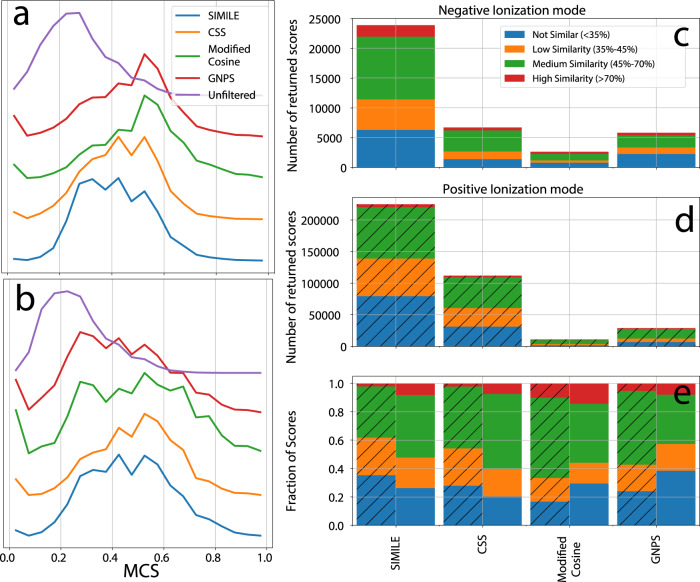
SIMILE identifies more pairs of spectra with meaningful structural similarity in comparison with Core Substructure Search (CSS), GNPS, and Modified Cosine using maximum common substructure (MCS) as a proxy for structural similarity. The inset histograms (**a** and **b**) show the distribution of MCS for unfiltered pairs of spectra and the MCS distribution for each algorithm in positive and negative ionization modes (respectively). The number of pairs with low, medium, or high structural similarity are shown for each algorithm in (**c** and **d**); and the fraction of similarity scores by each approach in (**e**) where positive ionization mode—dashed lines and negative ionization mode are solid bars.

Since each pair of spectra is accompanied by a pair of corresponding chemical structures, for each ionization mode, we performed an all-vs-all calculation across all pairs to calculate the maximum common substructure (MCS) Jaccard similarity coefficient. MCS is a property of a pair of molecules computed based on their overlapping chemical structure. If you know the chemical structure of each molecule in a pair of molecules, then the MCS can be easily calculated. This was used to define a rubric of chemical similarity in which an MCS less than 0.35 is considered “not similar”, between 0.35 and 0.45 to be “low similarity”, 0.45–0.7 to be “medium similarity”, and greater than 0.7 to be “high similarity”. These cutoffs are based on the finding that, across all pairs of molecules, the average MCS plus one standard deviation was 0.4. The distribution of MCS values across unfiltered pairs of molecules can be seen in Fig. 3a, b.

On all pairs of spectra, we then performed an all-vs-all calculation using a range of algorithms, including the MatchMS implementation of modified cosine, SIMILE, GNPS-cosine scoring, and Core Structure-based Search (CSS)^3,5,12,21^. Using the MCS associated with each pair of spectra/compounds as the ground-truth structural similarity for each pair, we sought to test how well each algorithm could convey structural similarity from spectral similarity. The absolute (Fig. 3a, b) and relative number (Fig. 3c) of returned pairs are shown in Fig. 3, and for each similarity category, the actual number of pairs can be seen in Supplementary Table 2. These both show that SIMILE finds the largest number of associations across all similarity categories.

The parameters chosen for each spectral similarity algorithm are such that an experienced practitioner would be comfortable choosing them. In addition, each algorithm returned approximately the same percent of “not similar”, or false positive, and one can see that the algorithms all have comparable abilities to return a relative number of structurally similar results. As can be seen in Fig. 3c, the fraction of “not similar” (blue) hits ranges between 20 and 30% of total hits. For SIMILE and CSS, there is a slight improved performance in negative ionization mode (~20% “not similar” in negative vs. 30% in positive), but for modified cosine, there is a slight improvement in performance for positive ionization mode. In the unfiltered comparisons shown in Fig. 3a, the amount of “not similar” pairs of spectra comprises approximately 80% of the pairs, demonstrating that all algorithms greatly enhance the recovery of structurally similar pairs of molecules from tandem mass spectra.

**Fig. 4:**
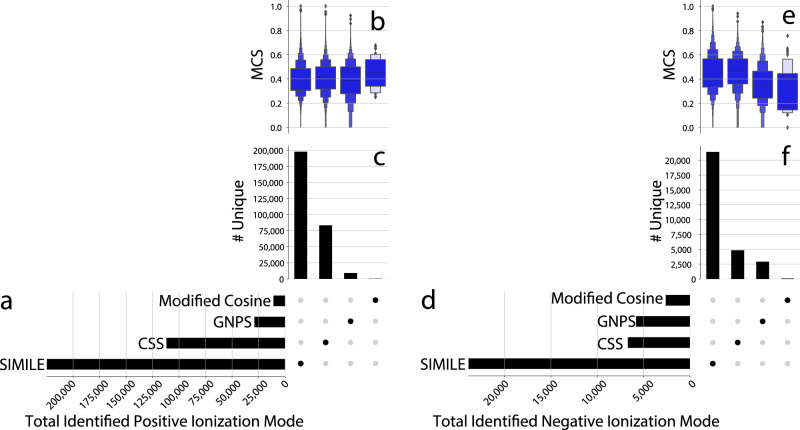
SIMILE identifies the largest number of similar pairs and 88% and 89% of the pairs of spectra identified by SIMILE in positive and negative ionization mode were not found by any other algorithm. Pairs of spectra identified by each algorithm for positive (**a**–**c**) and negative (**d**–**f**) ionization modes can be seen in these UpSet plots. The total number of pairs identified by each algorithm is shown in the bar chart to the left (**a** and **d**), and the unique pairs identified by each algorithm along with their corresponding structural similarity are shown in (**b**, **c**) and (**e**, **f**). Boxen plots (**b**, **e**) show several quantiles of the maximum common substructure (MCS) for each algorithm to approximate a distribution^38^.

Using these parameters for each algorithm for this analysis, SIMILE was found to identify more structurally similar pairs than all other algorithms in positive and negative ionization modes (Fig. 3c, d). More importantly, the unique and intersecting pairs of spectra that each algorithm identifies (Fig. 4) show that the algorithms yield nonoverlapping pairs of spectra. This implies that without sacrificing quality, a combination of these tools would likely yield the best results. To focus on highly similar pairs, (MCS > 0.7, Supplementary Figs 3, 4), a confusion matrix can be seen that shows the specific true-/false-positive and true-/false-negative counts for each algorithm. These algorithms all have comparable precision, but SIMILE was found to match more true positives.

To further understand the degree to which SIMILE can find more pairs of structurally related molecules, a synthetic dataset was created consisting of 20 flavonoids and 20 indoles randomly selected from the Berkeley Lab public GNPS reference library. Molecular networks were created using either GNPS or SIMILE scoring (Supplementary Fig. 5a, b). Because these classes of molecules have a conserved core structure, one can reliably identify moieties of each molecule that are present/or absent in another. For the ninety-seven pairs of flavonoids that were found by SIMILE and not found by GNPS, their aligned structures show multiple modifications (Supplementary Fig. SI5 and Supplementary Dataset 1).

**Fig. 5:**
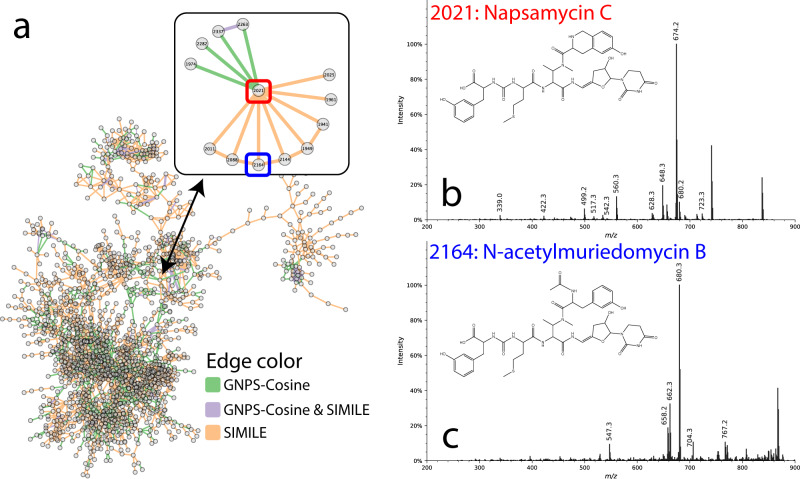
Starting with a spectrum from node 2021, which was identified as Napsamycin C from traditional molecular networking work, SIMILE found additional connections that would have been missed if only cosine scoring was used. The network shown in **a** contains edges from SIMILE, GNPS-Cosine, and both techniques. The connection to the spectrum from node 2021 shown in **b** was connected to the spectrum from node 2164 shown in **c**. Both spectra closely matched the spectra described for these compounds from published work (matching ions are shown)^22,23^. The cosine score between these two spectra is near zero.

To test the validity of using SIMILE for a real-world application to molecular networking, we searched all pairs of spectra from a publicly available dataset of chemical extracts of *S. roseosporus* NRRL 15998 and *Streptomyces sp*. DSM5940. Scoring these pairs with both GNPS-cosine scoring and SIMILE, we sought to determine if SIMILE could help gain additional information regarding the relationships between the spectra. Following the traditional scoring and network pruning approaches used in GNPS, many additional edges were identified by SIMILE, which could be explored (Fig. 5a). Focusing on the previously identified molecule, Napsamycin C, several connections (Fig. 5a inset) were found by SIMILE and not GNPS and vice-a-versa^22^. An example demonstrating additional insight from SIMILE, is a connection not observed before that links Napsamycin C to N-acetylmureidomycin B, another structurally related compound^23^. The fragmentation data shown in Fig. 5b, c match that described previously for these compounds^22,23^. Because the GNPS-cosine score was near to zero, this connection would have been missed by traditional molecular networking approaches and enabled putative annotations that were previously not possible.

Since this is the first application of a pair-specific similarity matrix for fragmentation spectra, we sought to better understand examples where SIMILE performed remarkably well and where SIMILE performed poorly. To look closely at cases that illustrate certain strengths and weaknesses of the current implementation of SIMILE, two cases were selected and shown in Fig SI6. Shown in Fig SI6a is a case comparing spectra from two flavonoid molecules with high structural similarity (MCS of 0.89) but differing by four relatively small non-stereo-specific structural differences. In this example, there is a spectrum with 26 ions from kaempferol 3-O-xyloside and a spectrum with 18 ions from mearnsitrin. The alignment identified by SIMILE follows a nearly unbroken path using 16 out of a possible 18 fragment ions, with an alignment score of 0.84 and a *p*-value of 0.008. In line with the view that modified cosine can struggle to align compounds with multiple structural differences, modified cosine only aligned 4 ions and had a score near zero. This example demonstrates a case where a precursor ion neutral-loss shift between the two spectra fails to align the spectra, but traversing the pair-specific similarity matrix with a dynamic programming approach yields an excellent alignment.

Shown in Fig SI6b is a case comparing the spectra from two secondary bile acids (deoxycholic acid with 19 ions and alpha-muricholic acid with 18 ions) differing by two structural modifications: the addition of a hydroxyl group and the relocation of another hydroxyl group. This pair of molecules has an MCS of 0.91. Like the example above, these spectra come from compounds with high overall structural similarity and differ by more than one structural difference. However, in this case, SIMILE failed to find reliable alignment. Only 6 out of 18 ions were aligned with an alignment score of 0.22 and a *p*-value of 1. By comparison, modified cosine aligned 7 ions, including the top-two ions, resulting in a score of 0.84. As can be seen in the similarity matrix, there are two potential paths. One path shifted by the precursor ion neutral loss and one path based on *m/z* differences of the fragment ions. Likely, an improvement to the SIMILE algorithm that incorporates precursor ion neutral-loss differences (like modified cosine) would aid this case and potentially many others.

## Discussion

Spectral similarity applications, like spectral networking, benefit from increasing connections between truly similar metabolites. One way of accomplishing this is by using a scoring method that is robust to arbitrary structural differences and provides statistical significance. In protein-sequence alignment, this is achieved by using substitution matrices with alignment algorithms that generate scores according to known distributions^24–26^. However, substitution/similarity matrices also appear in many other contexts, including text, speech, video, or even mathematically abstracted signals^27–30^. Once a similarity matrix is chosen, widely known methods can be used to calculate optimal alignments between pairs of signals^16,31,32^. To this end, we have developed a method of generating similarity matrices for fragmentation spectra to associate fragment ions by the similarity of their fragmentation paths. We found that our alignment method, SIMILE, yields different and typically more associations than cosine-based scoring algorithms and significant alignment scores generated by SIMILE correspond to compound structural similarity. SIMILE is currently limited to analysis of protonation/deprotonation, which is certainly a limitation. We see that developing approaches to comprehensively addressing additional adducts is an important direction for future research.

As can be seen in Figs. 4, 5, SIMILE identifies different pairs of compounds than other algorithms. This provides evidence that the SIMILE alignment scores and *p*-values are capturing aspects of how similar compounds fragment similarly. For modified cosine, the number of matching ions acts as a heuristic to approximate the significance of aligning/scoring the similarity of two spectra; in contrast with SIMILE, there is a mechanistic calculation of significance using a framework based on fragment ion substitutability. Likely, there will be classes of molecules that are more appropriate for cosine-based scoring than SIMILE-based scoring. Consequently, it makes sense to use SIMILE as an algorithm to accompany traditional scoring approaches (like modified cosine).

The underlying SIMILE distance measure for comparing fragment ions is closely related to Euclidean Commute Time Distance (ECTD), which has the property of decreasing with the number of connecting paths and increasing with the “length” of connecting paths^33^. The number of paths connecting two fragment ions increases when the number of total fragment ions increases. Likewise, the “length” of a path connecting fragment ions decreases when the frequency of m/z differences in the path increases. In other words, two fragment ions are similar if they are connected by many paths exhibiting high- frequency *m/z* differences.

Saerens et al. prove that the pseudoinverse of the graph laplacian acts as a covariance matrix with respect to ECTD^33^. We use a normalized and directed graph laplacian as the SIMILE similarity matrix to ensure that similarity scores in the fragment ion similarity matrix are normalized. We can then score fragmentation-spectra pairs by using a dynamic programming algorithm to align their fragment ions according to similarity scores^32^. This in turn mirrors how pairs of protein sequences can be scored by aligning their residues according to a substitution matrix.

For much of metabolomics research (including spectral-based molecular networking), it is necessary to use intermediate spectral similarity scores. Interrelating fragmentation spectra with intermediate scores in SIMILE is aided by a significance estimate; without statistical significance, intermediate similarity scores are to some degree uninterpretable. For example, intermediate scores could imply moderate structural similarity or tentative high similarity. Interpretation of intermediate scores has a direct effect on how researchers prioritize metabolites of interest and ultimately on the outcome of their research. As such, SIMILE significance estimation assists in deriving biological insight from metabolomics data when confronted with underexplored biochemistry. The significance of SIMILE alignments is calculated via a Monte Carlo permutation test with alignment score as the test statistic. It is important to note that significant SIMILE alignment of fragmentation spectra does not necessitate compound structure similarity. This is similar to how protein sequences can have significant alignment but yield different tertiary structures or functions. Likely, a significant SIMILE alignment indicates that two molecules fragment similarly, not that they necessarily have similar structures. Nevertheless, the results presented here show that significant fragmentation-spectra alignments often correspond to moderate-to-high structural similarity.

While SIMILE is a very promising approach for scoring pairwise spectral alignments, since this is the first application of scoring metabolite-fragmentation spectra using a similarity matrix, close attention must be applied. We recommend complementing SIMILE with the use of cosine-based scoring for compound identification. There is a massive amount of literature on cosine-based scoring, is well established, and has been in use for many decades. Since we find that SIMILE often works for cases where cosine-based scoring fails, using both approaches will provide more identifications. In addition, SIMILE provides a *p*-value and an alignment between fragments.

This said, it is likely that the parameters used in this study to compare alignment algorithms could be further optimized to improve performance. While beyond the scope of this work, this would likely provide additional insights on how the various approaches can be best used as part of metabolomics workflows. However, such optimizations almost certainly result in trade-offs in performance, for example, improving specificity vs. number of hits, both of which can be desirable objectives. In addition, from the m/z differences in the alignment, one can glean structural clues regarding the differences between the molecules. For example, the predominant *m/z* difference in Fig SI6a of 30.983 likely corresponds to [+O2 −H]. This is not surprising, given that one of the chemical differences between the two structures is the addition of two oxygens. We see this methodology being useful for elucidation of novel natural products by using the fragmentation spectra of known members of the same chemical class as “scaffolds.” In addition, as shown in Fig SI6b, there are improvements to the SIMILE algorithm that can likely boost its reliability and interpretability.

Here we describe SIMILE, a metabolomics tool immediately useful to complement existing widely used approaches with the potential to open up a completely new area for research in fragment ion similarity matrices with significance estimation. This approach provides a scoring and significance framework for discovering relationships between molecules that would have been dismissed with existing approaches. As such, using SIMILE as an algorithm to accompany traditional scoring approaches (like modified cosine) should lead to increased discovery in multiple fields, including compound and pathway discovery, and other useful applications of spectral networking.

## Methods

### Overview

The SIMILE algorithm calculates spectral similarity by aligning substitutable fragments, identification of the significance of their alignment; and scoring the degree to which two spectra are correlated. The underlying mathematical framework for these calculations is described below. Python code for each step is available in the GitHub repository (https://github.com/biorack/simile) as the release “v1_manuscript-submission”. Figure 2 illustrates the SIMILE algorithm with two hypothetical molecules. SIMILE can be evaluated interactively alongside other scoring algorithms in the GNPS Similarity Hub (example result for SIMILE scores and *p*-values calculated—Hub Link) where spectra from [M + H] + adducts for N-acetyl-5-hydroxytryptamine and 5-hydroxy-DL-tryptophan are scored.

The fragmentation process in tandem mass spectrometry can be modeled as a network of fragmentation paths connecting precursor ions to product ions with fragmentation reactions^34^. In addition, it is broadly assumed that similar structures fragment similarly. If this assumption holds, a similarity measure for fragment ions, which considers their fragmentation paths ought to be a good proxy for their structural similarity and, when aggregated, the similarity of the intact structures. To find such a similarity measure, we rely on the following observation: a modification to a molecule can add and/or remove fragmentation reactions to fragmentation paths. For modified fragmentation paths that do not remove fragmentation reactions, there will be a one-to-one substitution from the original fragment ions to modified fragment ions. The *m/z* differences cross-linking such fragmentation paths will be characteristic of the modification and their frequency proportional to the similarity of the fragmentation paths.

Because we do not know fragmentation paths for fragment ions in general, we consider the frequency of all pairwise *m/z* differences. While the frequency of *m/z* differences alone suffices in providing a “shortest path” distance between fragment ions, it remains insensitive to fragmentation paths. After all, a single common *m/z* difference between two fragment ions may very well be spurious. Instead, we are interested in consistent *m/z* differences cross-linking the same sets of fragment ions that are more likely to result from similar fragmentation paths.

For this reason, we use the average “commute time” of fragment ions as our distance measure that considers all paths weighted by *m/z* difference frequencies. Specifically, SIMILE works by projecting fragment ions into a Euclidean commute time distance (ECTD) space dependent on the frequency and connectivity of the *m/z* differences between them.

We rely on the variance–covariance matrix with respect to ECTD space for the remainder of the SIMILE algorithm because it forms a valid kernel (i.e., similarity function in the strict mathematical sense), which we denote the SIMILE similarity matrix. When comparing fragmentation spectra A and B, it is important to note that intraspectral (i.e., A vs. A) and interspectral (i.e., A vs. B) comparisons are considered in tandem by forming a square (A + B) by (A + B) similarity matrix. This is critical, because our *p*-value exchanges fragment ions between the A and B and so the fragment ions must be projected into the same space.

In order to quantify the similarity of fragmentation spectra A and B, the SIMILE similarity matrix is used in conjunction with a dynamic programming algorithm related to those used for pairwise alignment of biological sequences. For the sum of the similarities in the alignment, we denote the SIMILE alignment score and use as a proxy for compound similarity.

While alternatives to *m/z* order-based alignment of fragment ions exist such as maximum weight matching (aka Hungarian algorithm or linear sum assignment optimization), there is a reason as to why one may prefer alignment. Because SIMILE projects fragment ions into a space dependent on the frequency of *m/z* differences and not the magnitude of those differences, it is possible for similar fragment ions to have very different masses. However, one would naturally expect large mass differences to imply comparatively less structural similarity than smaller mass differences. By using alignment instead of maximum weight matching, we are in effect forcing the score to address this trade- off albeit stochastically. While alignment is not guaranteed to appropriately match similar fragment ions (Fig. SI6b), we found alignment to perform well in predicting structural similarity.

Ideally, SIMILE would align fragment ions that are substitutions with respect to their fragmentation paths. We assume that substitutable ions are more similar to each other than to their precursor or product ions. If this were not the case, then such precursor ions could be considered substitutable with its product ions that break the one-to-one correspondence definition of substitutability. To this end, we construct a random permutation test for the robustness of the SIMILE alignment score to invasion by precursor/product similarity scores. If the observed SIMILE alignment score is consistently higher than those from the null distribution, then we cannot reject the aligned ions being substitutable. By default 10, 100, and 1000 iterations of the permutation are performed, and that early stopping occurs when the *p*-value does not improve by twofold.

### Compute time

While calculating the *p*-value is of time complexity O(knm) where there are k permutations of n-by-m similarity matrices, three key optimizations in the implementation details confer significant speedup by enabling vectorized operations. This effectively moves k times max(n,m) of the operations out of Python and into highly optimized compiled C code. One, because the gap penalty is zero and only the alignment score is needed (not the alignment), we can replace the O(nm) dynamic programming implementation for alignment with a single-loop iterating over min(n,m) in Python to greedily compute the alignment score using alternating vectorized maximum and rolling maximum operations. Two, the k-by-n-by-m array consisting of “stacks” of permuted similarity matrices is constructed by broadcasting the similarity matrix using k random permutation indices that minimize needless data copying. Three, because permuted similarity matrices are stacked, the alignment of each stack is independent of one another, and the underlying alignment-score operation is partially vectorized, we extend the vectorized alignment- score operation to operate on each stack in a vectorized fashion. On a modern Linux workstation and at a *p*-value of 0.0001, we found the average compute time to be 20 milliseconds for SIMILE comparisons within NIST20 without any parallelization (Figure SI2). For comparisons that have a very low SIMILE *p*-value, the computation can take up to 10 seconds, but this is extremely rare within the NIST20 comparisons. Early stopping can restrict *p*-value calculations to a *p*-value of 0.01 and shorten this calculation time by approximately a factor of 100. For spectra with 100 s of ions that align extremely well, the compute time can be even longer. This is a limitation, and would increase the time to calculate the all-by-all comparisons, for datasets rich in these types of spectra.

### Similarity matrix

Construction of a pair-specific *m/z* similarity matrix takes the concatenation of two ordered *m/z* lists from fragmentation spectra X of length *m* and Y of length *n* as input. The pairwise (outer) difference of the concatenated *m/z* list is stored in an *m* + *n* by *m* + *n m/z* difference matrix, **D**. (Fig. 2a). The *m/z* difference matrix **D** is delineated by four quadrants: X minus X (e.g., all of the intraspectral differences in X) in the top-left m by m, Y minus Y (e.g., all of the intraspectral differences in Y) in the bottom-right n by n, X minus Y in the top-right m by n, and Y minus X (e.g., all of the interspectral differences between X and Y) in the bottom-left n by m. In Fig. 2, only the top-right quadrant is shown to illustrate *m/z* differences, frequencies of specific differences, and ion similarity scores comparing the two hypothetical molecules, but all four quadrants are used in the calculation. For each element **D**_ij_ of the top m by m + n quadrants of **D**, the *m/z* differences in the block that are within a given *m/z* tolerance window of **D**_ij_ are counted and element-wise assigned to the *m/z* difference frequency matrix, **C** (Fig. 2b). Repeat the procedure to fill the bottom n by m + n quadrants of **C** using the bottom m by m + n block of **D**.

To calculate a similarity matrix from the *m/z* frequency matrix, we used the approach described by Li et al.^20^. Dividing each row of the *m/z* difference count matrix **C** by the sum of the row yields the *m/z* transition matrix **T**. Each element **T**_ij_ describes the probability of the *i*^th^ *m/z* from the concatenated *m/z* list transitioning to the *j*^th^ *m/z*. As such, the transition matrix defines a Markov chain representation of the *m/z* values from fragmentation spectra X and Y. Let **I** be the m + n by m + n identity matrix, defined as having ones along the diagonal and zeros elsewhere. Let **p** be the stationary probability distribution of the *m/z* transition matrix **T**, defined by the property that **pT** = **p**. The stationary probability distribution **p** is calculated by dividing the principal eigenvector of **T** by its sum. The directed graph Laplacian **L** of **C** is defined as **p**^**1/2**^**(I**-**T)p**^**−1/2**^. Finally, the *m/z* similarity matrix **S** is calculated by taking the Moore–Penrose pseudoinverse of (**L** + **L**^**T**^**)/2. S**^**XX**^ and **S**^**YY**^ are defined as the top-left m-by-m quadrant and the bottom-right n-by-n quadrant of **S**, respectively. Likewise, **S**^**XY**^ and **S**^**YX**^ are defined as the top-right m-by-n quadrant and the bottom-left n-by-m quadrant of **S**, respectively.

### Alignment and alignment score

The alignment and alignment score are calculated using the dynamic programming approach described by Needleman and Wunsch for sequence alignments, but with a fixed gap penalty of zero^32^. It is defined by the recurrence relation

1 $$A(i,j)={{\max}}\left\{\begin{array}{c}{A(i-1,j-1)+{\mathbf S}_{{{{{{\mathbf{ij}}}}}}}}\\ {A(i-1,j)}\\ {A(i,j-1)}\end{array}\right.$$

with initial conditions *A*(*i*,0) = 0 for all *i* and *A*(0,*j*) = 0 for all *j*. The terminal value of *A* is the alignment score and tracing back the elements of **S**, Bhich contribute to this score, yields the alignment.

### Alignment significance

The significance of the alignment is calculated via a permutation test with the alignment score used as test statistic under the null hypothesis that *m/z* values are exchangeable between *m/z-*ordered fragmentation spectra X and Y only if they generate fragmentation spectra X′ and Y′ that are also *m/z* ordered. This null hypothesis corresponds to the assumption that exchanging interspectral similarity scores in **S**^**XY**^ and **S**^**YX**^ with intraspectral similarity scores in **S**^**XX**^ and **S**^**YY**^ improves alignment scores so long as *m/z* order is preserved in the resulting **S**^**X′Y′**^ and **S**^**Y′X′**^.

Rather than compute every valid permutation of **S** to calculate the *p*-value, we use Monte Carlo testing with early stopping to asymptotically approach the true *p*-value with bounded and known error^35^. In practice, this is done by first generating a random permutation of indices that index the concatenated *m/z* list, such that the first *n* entries are *m/z* ordered and the last *m* entries are *m/z* ordered, which implicitly generates hypothetical fragmentation spectra X′ and Y′. This permutation of indices is then used to permute the rows and columns of **S** symmetrically. The alignment score is calculated with the alignment algorithm described above. The *p*-value is then the probability that a random alignment score from the empirical distribution is greater than the observed alignment score or one out of the number of iterations, whichever is greater (Fig. 1h). A score cutoff greater than or equal to 0.7, a *p*-value cutoff less than or equal to 0.05, and a number of matching ion cutoff greater than or equal to 10 were used to identify similar spectra for the majority of calculations in this work.

### Validation and development dataset

The algorithm was developed and validated using tandem mass spectra from the commercially available electrospray ionization spectra available from the National Institute of Standards and Technology (NIST 2020) library. These spectra are acquired on a variety of instruments (Supplementary Table 1) under a variety of conditions. They were filtered to include only [M–H]^−^ adducts for negative-mode comparisons and [M + H]+ adducts for positive-mode comparisons, and the closest collision energy to 40 eV within 5 eV (SI Fig. 1). Many other collision energies are available for study, but we limited this to one specific value for the sake of defining a highly controlled set of comparisons. Future studies can extend to 0 V, 20 V, 40 V, 60 V, 80 V, and 100 V to identify the optimum collision energy for assessing similarity with tandem mass spectra. Each compound was assigned to a chemical class using the ClassyFire web service^36^. For each unique inchi key, a JSON file containing the chemical class was retrieved from the web service by the following URL http://classyfire.wishartlab.com/entities/ik.json where ik is the inchi key for the compound. While this JSON file provides predicted compound kingdom, superclass, class, and subclass, only the class was retained for this analysis.

### Spectral similarity algorithms

In general, the following score cutoffs for each algorithm are based on the published parameters. For CSS and SIMILE, the development teams worked together to select parameters for the comparisons. As is stated above, for SIMILE, a score cutoff greater than or equal to 0.7, a *p*-value cutoff less than or equal to 0.05 and a number of matching ion cutoff greater than or equal to 10 were used to identify similar spectra for all calculations in this work (with the exception of the Napsamycin molecular network). The MatchMS python package version 0.6.2 was used to calculate the modified cosine spectral similarity score and determine the number of matching ions^21^. Each spectrum was square-root intensity scaled and further normalized using the MatchMS function “normalize_intensities”. Pairs of spectra were evaluated with the modified_cosine.pair() function, a scoring algorithm that includes precursor loss masses and intensities. A score cutoff greater than or equal to 0.6 and a number of matching ions greater than or equal to 6 were used to identify similar spectra.

The Core Structure-based Search (CSS) algorithm was rewritten in C# for large-scale spectral comparison^5^. For each MS/MS spectrum, top-30 fragments (sorted by intensity) were reserved, and peak intensities were subject to normalization and square-root transformation. The maximum number of hypothetical neutral losses (HNLs) was set to 100, and the minimum HNL mass was set to 36. Both CSS score and CSS match number were output to optimize the cutoffs for similar spectra. A score cutoff greater than or equal to 0.6 and a match number cutoff greater than or equal to 40 was used to identify similar spectra.

GNPS-aligned cosine spectral similarity was written in C++ for large-scale comparisons and run externally of GNPS’ molecular networking to benchmark the scoring function in isolation of molecular networking. This was done specifically because the molecular networking workflows introduce other heuristic processing algorithms that increase the interpretability of the molecular networks that interfere with benchmarking. MS/MS spectra were aligned and scored with a fragment tolerance of 0.5 Da. A score cutoff greater than or equal to 0.6 and a number of matching ions greater than or equal to 6 were used to identify similar spectra.

### Molecular networking

Following the calculation of all-by-all SIMILE scores, filtering both by score cutoffs and topology is required to make an interpretable network. The all-by-all scores were filtered to remove scores less than 1.0 and *p*-value greater than 0.05 for SIMILE. This is a more strict threshold than was used in the NIST20 data. Finally, the network topology was constrained first to preserve only the 10 top-scoring edges and second to constrain subgraph size to 100 nodes or less. This resulting SIMILE network was merged with the existing network based on GNPS-cosine scores. The existing GNPS network was created using the same topological filters as were used for SIMILE.

### Maximum common substructure (MCS)

The chemical similarity metric used in this paper is the Jaccard similarity coefficient, *s*, of the overlapping bonds given by *s* = *N* / ((*N*_*A*_ + *N*_*B*_) − *N*) where *N* is the number of overlapping bonds between the pair of molecules *A* and *B*. *N*_*A*_ and *N*_*B*_ are the number of bonds in each of the molecules A and B, respectively. The alignment of a connected block between each pair of molecules is done using the RDKIT python package with a timeout of 1800 seconds and ringMatchesRingOnly set to False^37^. One pair of compounds timed out and was discarded from further analysis. Here we refer to the MCS Jaccard similarity of bonds as MCS.

### Reporting summary

Further information on research design is available in the Nature Research Reporting Summary linked to this article.

## Supplementary information

Supplementary Information

Reporting Summary

## Data Availability

Raw spectra for the work presented here came from three sources. For the majority of the analysis, the commercially available NIST20 Tandem Mass Spectral Library was used. Further analysis of molecular networking from Streptomyces isolates used a molecular network available in GNPS^3^. In addition, the flavonoid and indole comparison network was based on the publicly available Berkeley Lab GNPS library.

## Code availability

The SIMILE algorithm and example Jupyter notebooks are available on Github at https://github.com/biorack/simile/ and tagged as a release-labeled version 1.
