# Supplementary material for: SIMILE enables alignment of tandem mass spectra with statistical significance
Source: Nat Commun. 2022 May 6;13:2510. doi: 10.1038/s41467-022-30118-9 (PMC9076661; doi:10.1038/s41467-022-30118-9)
Supplement: Supplementary file 1 — Supplementary Information [file 41467_2022_30118_MOESM1_ESM.pdf]

SIMILE enables alignment of tandem mass spectra with statistical significance

Daniel G.C. Treen[†], Mingxun Wang[#], Shipei Xing[‡], Pieter C. Dorrestein[#], Katherine B. Louie[†], Tao Huan[‡], Trent R. Northen[†], and Benjamin P. Bowen[*†]

*To whom correspondence should be addressed: bpbowen@lbl.gov
[†]Environmental Genomics and Systems Biology Division &
The Joint Genome Institute
Lawrence Berkeley National Laboratory
One Cyclotron Road, Berkeley, CA, 94720

[#]School of Pharmacy and Pharmaceutical Science, University of California, San Diego, 9500 Gilman Drive, La Jolla CA 92093

[‡]Department of Chemistry, Faculty of Science, University of British Columbia, Vancouver Campus, 2036 Main Mall, Vancouver, V6T 1Z1 British Columbia, Canada

Supplementary table 1. Filtering of the NIST20 spectral library resulted in the following number of entries for each type of mass spectrometer and either protonated or deprotonated ions. Only one spectrum for each compound in each polarity was used for this study. As can be seen here, the majority of ions are from an orbitrap mass spectrometer resulting in high accuracy and high resolution reference spectra.

Instrument	adduct	compounds
Thermo Finnigan Elite Orbitrap	[M+H]+	13033
Agilent QTOF 6530	[M+H]+	1908
Orbitrap Fusion Lumos	[M+H]+	1804
Thermo Finnigan Velos Orbitrap	[M+H]+	480
Thermo Finnigan Elite Orbitrap	[M-H]-	7075
Orbitrap Fusion Lumos	[M-H]-	234
Agilent QTOF 6530	[M-H]-	129
Thermo Finnigan Velos Orbitrap	[M-H]-	8

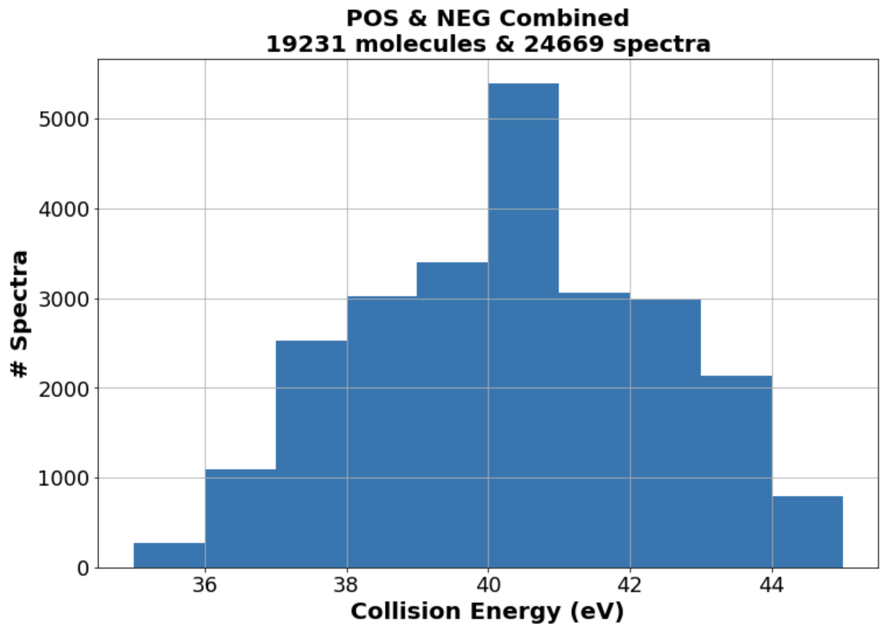

SI Figure 1. The closest spectrum to 40 eV for each [M+H]+ and [M-H]- spectrum in NIST20 high resolution spectra was selected. This subset provides a sizable amount of comparisons to make relations under controlled conditions. Within the NIST20 reference spectra, there are a wide range of collision energies used, but for the purposes of this study, only this narrow range was selected. Further studies could scan the collision energies available and identify optimum conditions for these types of comparisons.

Supplementary table 2. Raw counts of pairs identified by each algorithm and the unfiltered pairs for comparison. These are shown for both positive and negative ionization modes. Using the parameters discussed in the main text of this manuscript, the following counts of pairs of spectra were obtained. As can be seen below, each algorithm enriches the fraction of structurally similar pairs relative to the unfiltered distribution. Given a particular level of stringency required, one can easily calculate the confusion matrix from these values.

Scoring	Not Similar (<35%)	Low Similarity (35%-45%)	Medium Similarity (45%-70%)	High Similarity (>70%)	Ionization Mode
SIMILE	79549	59463	81004	4717	Positive
CSS	31304	29433	48746	2466	Positive
Modified Cosine	1849	1832	6225	1110	Positive
GNPS	6977	5423	15040	1523	Positive
Unfiltered	10967869	2164783	1266372	17322	Positive
SIMILE	6286	5130	10520	1934	Negative
CSS	1370	1292	3543	497	Negative
Modified Cosine	774	382	1086	376	Negative
GNPS	2238	1093	2019	459	Negative
Unfiltered	4886737	834219	522124	10481	Negative

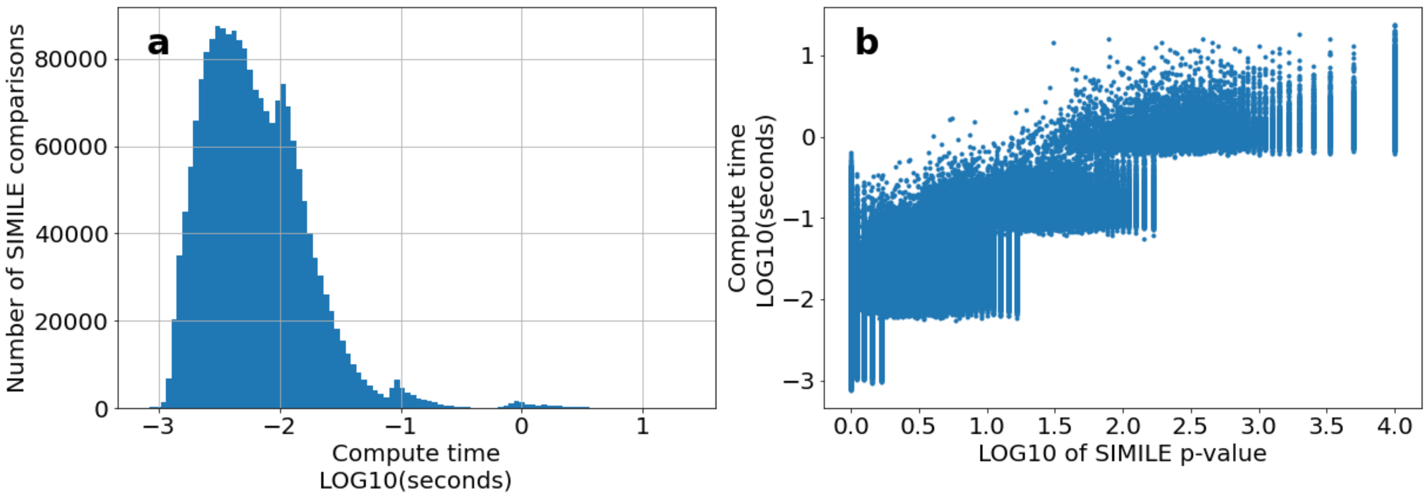

Figure SI 2. SIMILE has a mean compute time of 21 milliseconds without any parallelization **(a)**. For comparisons that have a very low SIMILE p-value, the computation can take up to 10 seconds, but this is extremely rare. Early stopping can restrict p-value calculations to a p-value of 0.01 and shorten this calculation time **(b)**. The time required for p-value and score calculation depends strongly on the number of associated ions as well. Comparisons between a pair of spectra that have high similarity take much longer than comparison between two dissimilar spectra. Consequently, certain datasets will take longer to score than others.

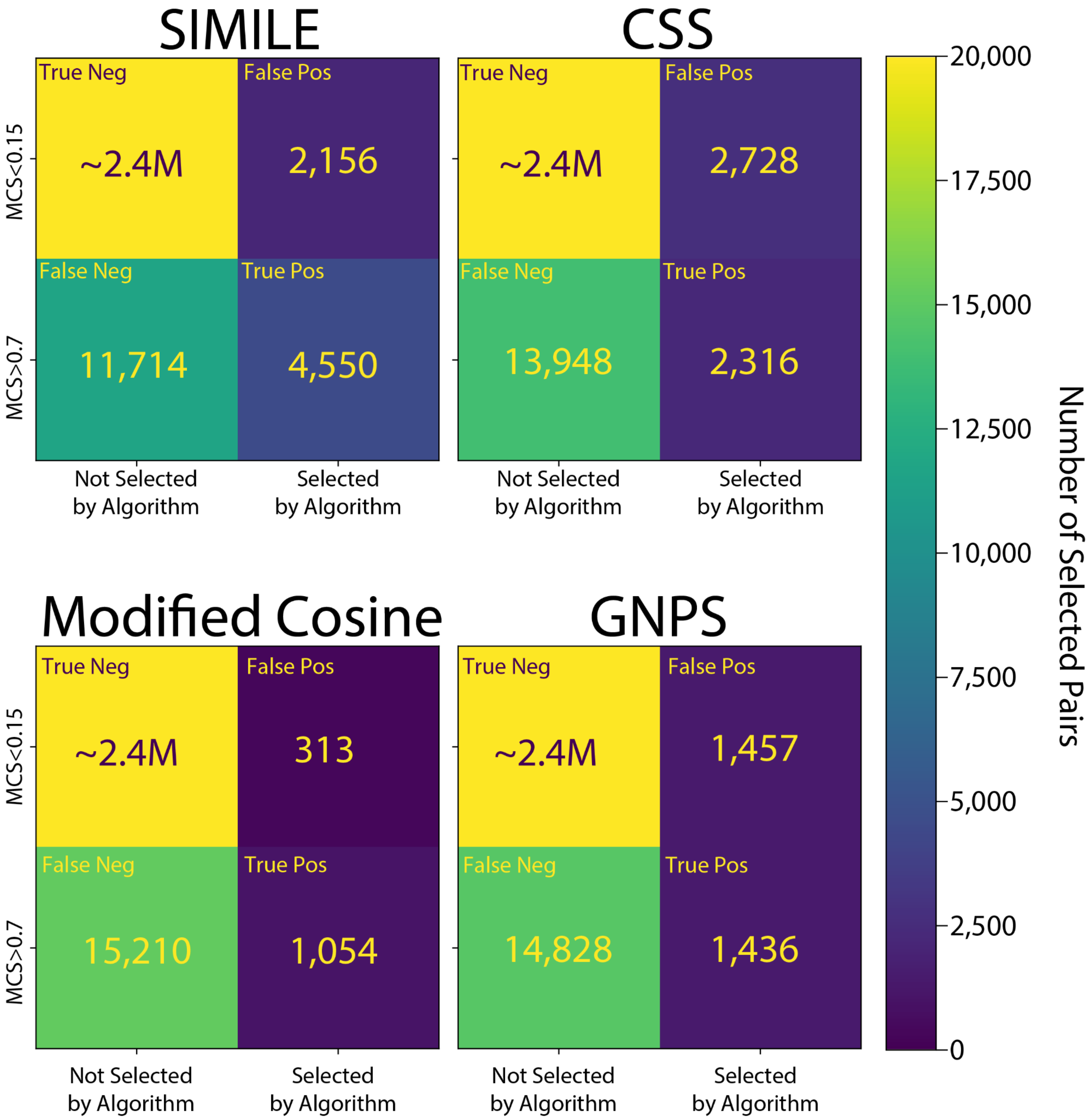

Figure SI 3. For spectra collected in positive ionization mode, this confusion matrix, where the most similar structures by maximum common substructure (MCS>0.7) are considered “similar pairs of structures” and MCS less than 0.15 is considered “not similar”, shows that for these parameters, these algorithms have comparable precision, but SIMILE identifies more pairs that are structurally similar.

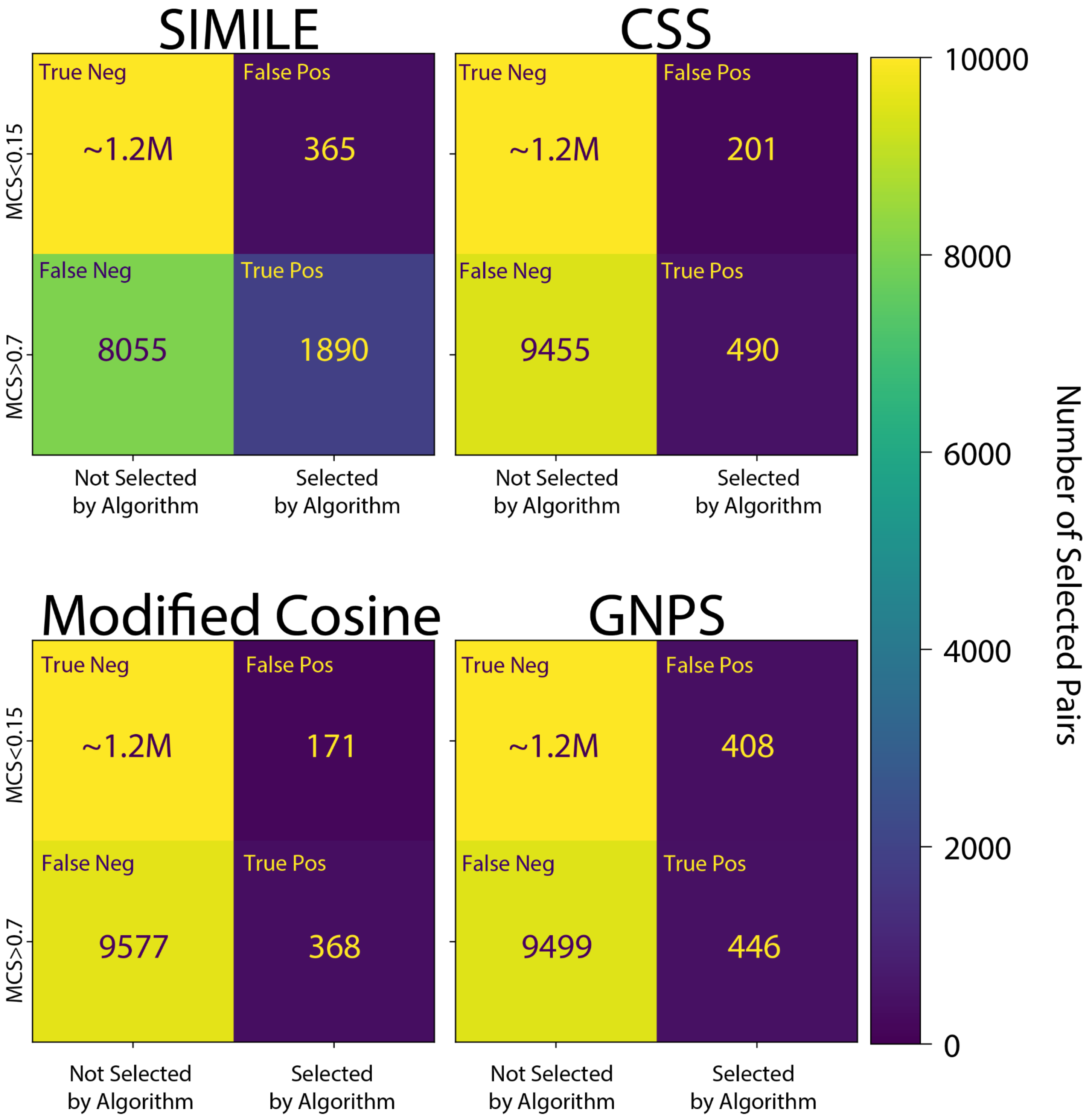

Figure SI 4. For spectra collected in negative ionization mode, this confusion matrix, where the most similar structures by maximum common substructure (MCS>0.7) are considered "similar pairs of structures" and MCS less than 0.15 is considered "not similar", shows that for these parameters, these algorithms have comparable precision, but SIMILE identifies more pairs that are structurally similar.

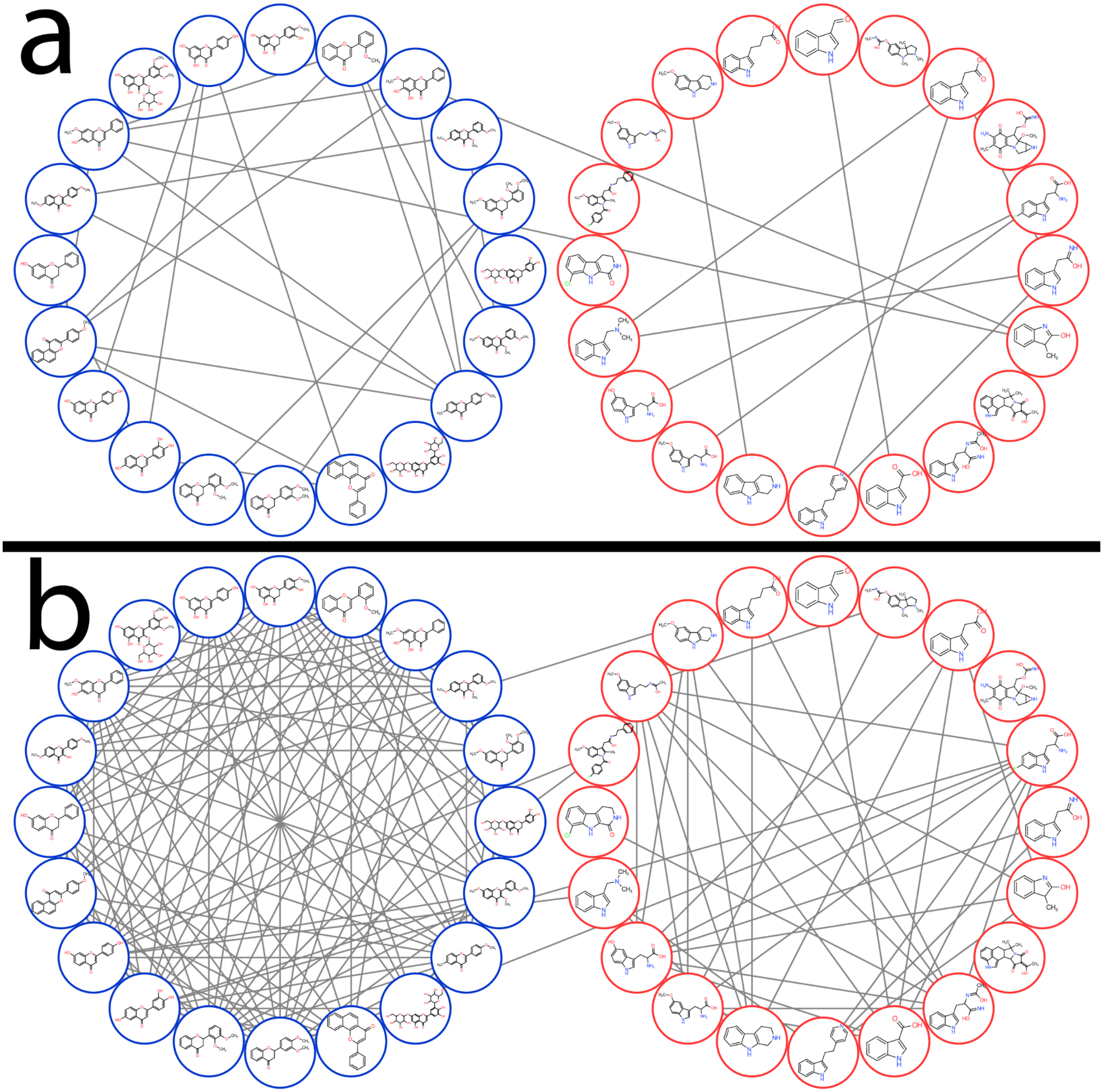

Figure SI5. Spectra from the Berkeley Lab GNPS reference library using Classyfire class attributes combining 20 flavonoids and 20 indole molecules chosen at random was analyzed using both GNPS scoring: cosine score > 0.5 in (a) and SIMILE scoring: $p<0.05$ in (b). These results were computed using the GNPS molecular networking platform. GNPS scoring is available here, and SIMILE scoring is available here. Red circles are indoles and indole derivatives; whereas blue circles are flavonoids. As can be seen, SIMILE associates more structurally related compounds while maintaining approximately the same false discovery rate between these two classes of compounds. Of the 190 potential flavonoid connections, 123 of them were found by either SIMILE or GNPS. SIMILE identified 121 whereas GNPS scoring identified 26: leaving two pairs found by GNPS and not SIMILE. The chemical structures corresponding to the 97 pairs of flavonoids found by SIMILE and not found by GNPS can be seen in a separate supplementary PDF. Not surprisingly, the majority of these pairs have multiple chemical differences that are not collocated on each molecule.

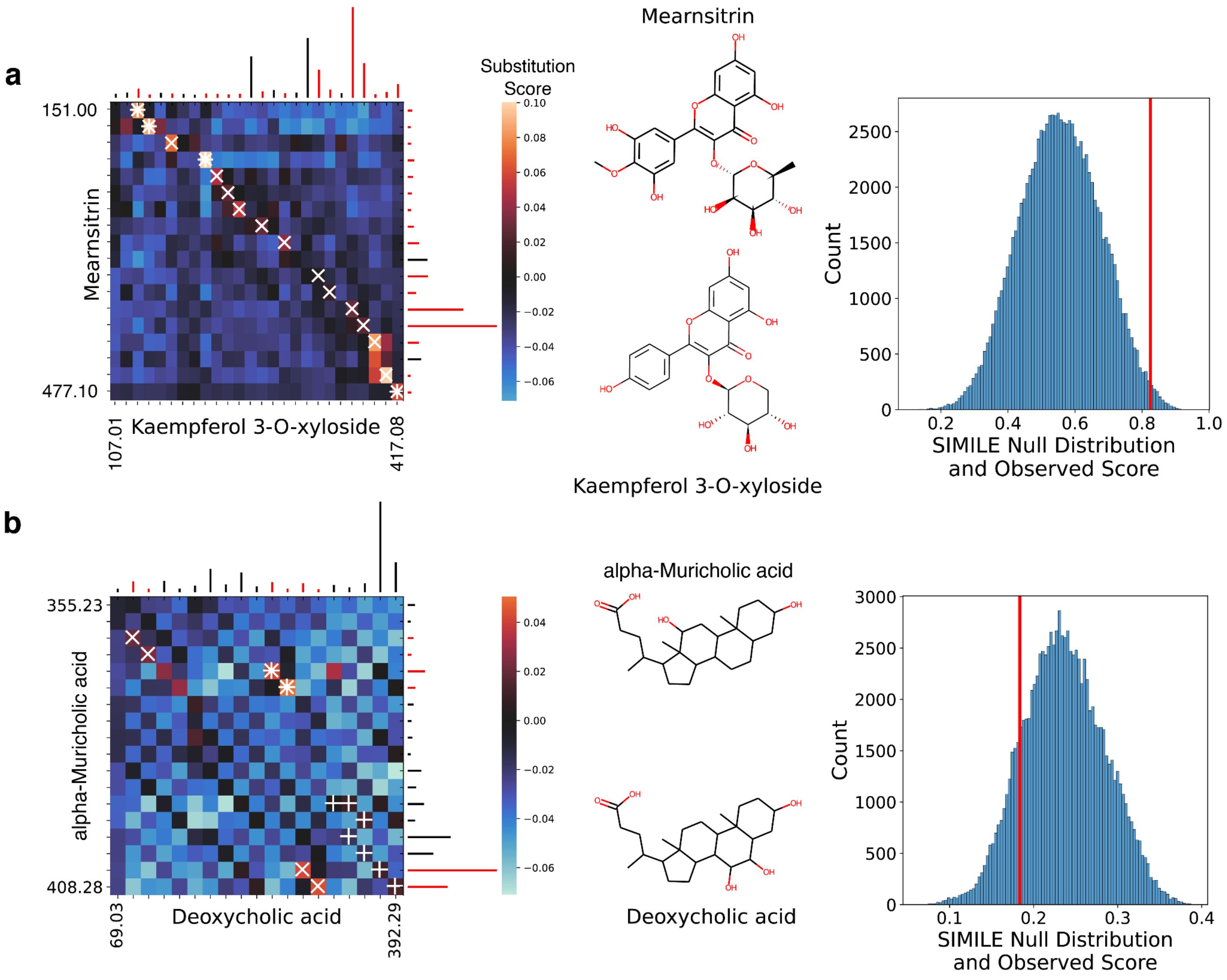

Fig. SI6. Two cases showing the detailed aspects of SIMILE alignment and scoring. Fragment ions aligned by both SIMILE and modified cosine are denoted by ✱; only aligned by SIMILE as ✖; and only aligned by modified cosine as ➕. Fragment ions identified by SIMILE are shown in red alongside the substitution matrix. The *m/z* differences in SIMILE alignments can correspond to compound structural differences unobservable by only considering differences in precursor ion neutral-losses. Alternatively, SIMILE alignments may underperform modified cosine when the optimal alignment relies on precursor ion neutral-losses. **a)** For a case where SIMILE dramatically outperforms modified cosine, none of *m/z* differences correspond to precursor ion neutral-losses and so the difference in the number of matching ions is extreme (4 vs 16). Additionally, 11 of aligned fragment ions have an *m/z* difference of 30.983 which likely corresponds to a chemical difference of [+O2 -H]. This pair has a SIMILE score of 0.84 and a p-value of 0.008. **b)** For a case where modified cosine dramatically outperforms SIMILE, a precursor ion neutral-loss alignment yields more matching fragment ions than SIMILE (7 vs 6). Additionally, SIMILE does not align the highest intensity ions together whereas modified cosine does. This pair has a SIMILE score of 0.22 and a p-value of 1.

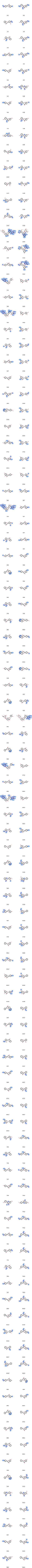
0-2
0-9
1-5
1-9
2-6
2-9
3-7
3-9
4-8
4-9
5-2
5-20
6-8
6-20
7-9
7-20
8-12
8-20
9-13
9-20
10-16
10-20
11-17
11-20
12-18
12-20
13-19
13-20
14-3
14-14
15-9
15-14
16-12
16-14
17-1
17-16
18-2
18-16
19-3
19-16
20-5
20-16
21-6
21-16
22-7
22-16
23-8
23-16
24-9
24-16
25-10
25-16
26-11
26-16
27-12
27-16
28-13
28-16
29-15
29-16
30-2
30-7
31-3
31-7
32-5
32-7
33-6
33-7
34-2
34-8
35-4
35-8
36-5
36-8
37-6
37-8
38-5
38-10
39-6
39-10
40-7
40-10
41-8
41-10
42-2
42-5
43-4
43-5
44-1
44-3
45-2
45-6
46-4
46-6
47-1
47-15
48-2
48-15
49-3
49-15
50-5
50-15
51-6
51-15
52-11
52-15
53-4
53-18
54-5
54-18
55-6
55-18
56-7
56-18
57-8
57-18
58-11
58-18
59-12
59-18
60-15
60-18
61-16
61-18
62-4
62-17
63-5
63-17
64-6
64-17
65-7
65-17
66-8
66-17
67-11
67-17
68-12
68-17
69-16
69-17
70-5
70-12
71-6
71-12
72-7
72-12
73-8
73-12
74-9
74-12
75-11
75-12
76-2
76-19
77-5
77-19
78-6
78-19
79-7
79-19
80-8
80-19
81-13
81-19
82-16
82-19
83-17
83-19
84-18
84-19
85-2
85-4
86-2
86-11
87-4
87-11
88-5
88-11
89-6
89-11
90-8
90-11
91-4
91-13
92-5
92-13
93-6
93-13
94-7
94-13
95-8
95-13
96-9
96-13
